# The mouse pubic symphysis: a narrative review

**DOI:** 10.3389/fphys.2025.1497250

**Published:** 2025-03-26

**Authors:** Ning Wang, Xue Tong, Yi-kai Li

**Affiliations:** ^1^ School of Traditional Chinese Medicine, Southern Medical University, Guangzhou, China; ^2^ School of Chinese Medicine, Hong Kong Baptist University, Kowloon, Hong Kong SAR, China; ^3^ The Third Affiliated Hospital, Southern Medical University, Guangzhou, China

**Keywords:** pubic symphysis, pregnant, postpartum, remodeling, animal model

## Abstract

Remodeling and relaxation of the mouse pubic symphysis (PS) are responsible for separating the pubic bone, allowing the passage of the full-term fetus, and ensuring safe delivery. PS in *postpartum* mice can rapidly return to a similar non-pregnant state, providing mechanical stability for the reproductive tract. During pregnancy and *postpartum* recovery, PS changes in mice are involved in many aspects, including extracellular matrix (ECM), matrix metalloproteinases (MMPs), cell phenotypes, hormones, and immune cells. The changes in PS in mice during pregnancy and *postpartum* convalescence were reviewed, and the possible mechanisms were discussed. We hope to attract more research interest to explore the biological mechanisms of this process better.

## Graphical Abstract

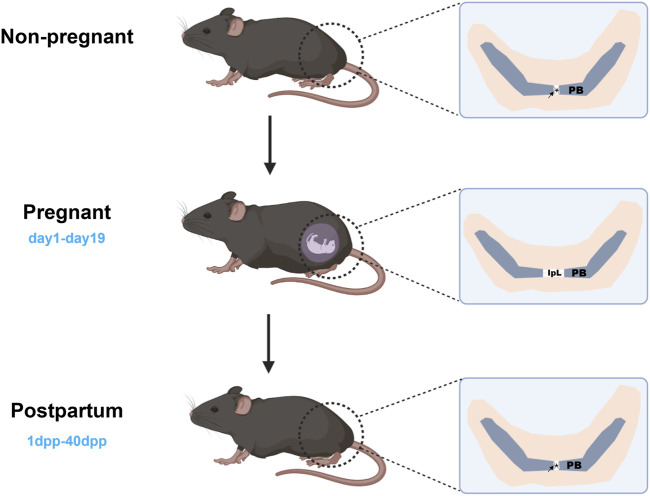

## 1 Introduction

In mammals, the pubic symphysis (PS) primarily comprises hyaline cartilage or fibrocartilage, depending on the species (Ruth, 1932). In guinea pigs (Wahl et al., 1977), mice (Ortega et al., 2003), bats (O'Connor et al., 1966), and humans (Crelin, 1969), PS is connected by fibrocartilage and can be remodeled into the interpubic ligament (IpL) during pregnancy (Ortega et al., 2003; Becker et al., 2010; Ruth, 1937). In contrast, PS in rats is connected by hyaline cartilage, and IpL is not formed during pregnancy (Ortega et al., 2003). During growth and development, PS in some species may transition from hyaline cartilage to bone through endochondral ossification, leading to synostosis (Ruth, 1936; Ruth, 1935). Overall, the classification and variation of PS joints are related to the species, reproductive mechanisms, and developmental stages (Ortega et al., 2003).

**FIGURE 1:**
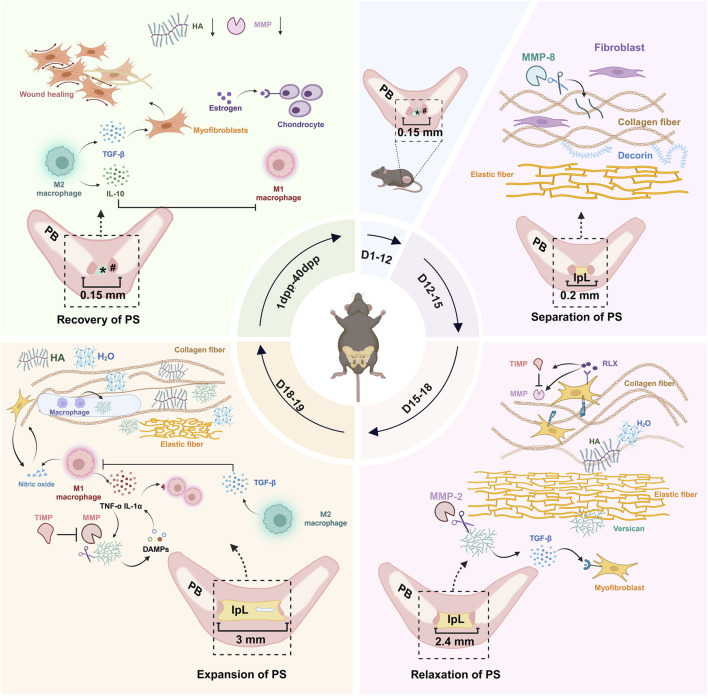
Dynamic remodeling of the mouse PS during pregnancy and *postpartum*. During pregnancy, the microstructure of the PS in pregnant mice remains highly consistent with that in non-pregnant mice from D1 to D12. Between D12 and D15, the PS begins to separate, and the formation of IpL occurs. Fibroblasts proliferate, and MMP-8 activity increases, contributing to ECM remodeling. Decorin plays a role in stabilizing collagen fibers. From D15 to D18, the IpL expands from 0.2 to 2.4 mm. This stage involves fibroblast proliferation, collagen fiber decomposition, untwisting, and dispersion, along with the distribution of water molecules attracted by versican and HA in the tissues. By D19 (delivery day), the IpL reaches approximately 3 mm, and pseudo-cavities appear within the IpL. These changes are linked to collagen fiber decomposition and reorganization, increased ECM hydration, macrophage activation, and upregulation of nitric oxide. From 1 dpp to 40 dpp, the IpL disappears, a fibrocartilage disc appears, and the PS returns to a structure similar to that of non-pregnant mice. #: hyaline cartilage; *: disc of fibrocartilage; PS, pubic symphysis; IpL, interpubic ligament; PB, pubic bone; MMP, matrix metalloproteinase; TIMP, tissue inhibitor of metalloproteinases; TGF-β, transforming growth factor-beta; TNF-α, tumor necrosis factor-alpha; IL-1α, Interleukin-1 alpha; IL-10, Interleukin-10; HA, hyaluronic acid; DAMPs, damage-associated molecular patterns; RLX, relaxin; dpp, days *postpartum*; ECM, extracellular matrix. (figure was created with Biorender.com).

In non-pregnant female mice, the articular surface of the pubic bone is capped with hyaline cartilage, united by a fibrocartilaginous disc, and supplemented by a connective tissue capsule surrounding the joint (Ortega et al., 2003). During mouse pregnancy, PS is fully expanded to meet delivery requirements, and this separation is attributed to three factors: (a) progressive reabsorption of PS; (b) swelling of the cartilage matrix; and (c) formation of IpL (Ortega et al., 2003; Storey, 1957). This process needs to be completed briefly to meet childbirth needs. At 3 days *postpartum* (3dpp), there is a notable decrease observed in the interpubic articulation gap (Consonni et al., 2012a), 5dpp PS has fibrocartilage characteristics (Veridiano et al., 2007), 10dpp cartilage cap is restored (Castelucci et al., 2018), and 40dpp PS returns to a similar non-pregnant state (Consonni et al., 2012b). The changes in mouse PS during pregnancy and *postpartum* recovery are strongly dramatic. Current studies have focused on the remodeling and relaxation of PS during pregnancy in mice, mainly involving extracellular matrix (ECM), cell phenotypes and morphology, and immune cells. These changes are associated with matrix metalloproteinases (MMPs), nitric oxide (NO), and relaxin (RLX) (Storey, 1957; Consonni et al., 2012a; Veridiano et al., 2007; Moro et al., 2012; Parry et al., 2005; Rosa et al., 2011). In this review, we tried to provide an overview of mouse PS changes during pregnancy and *postpartum*. According to the different time segments, we reviewed mouse PS’s complex change (Table 1) during pregnancy and *postpartum*. We focused on the remodeling and relaxation mechanisms of PS in mice during mid-to-late gestation and summarized the related mechanisms of *postpartum* PS recovery, contributing to our understanding of the biological mechanisms of PS during pregnancy and *postpartum* (Figure 1).

**TABLE 1:** Changes of the PS in pregnant and *postpartum* recovery mouse.

Time	Changes	Change-related mechanism	References
D1-12	—	—	Consonni et al. (2012a), Veridiano et al. (2007), O'Byrne and Steinetz (1976), Linck et al. (1975)
D12-15	IpL forms	Fibroblasts proliferate	Veridiano et al. (2007), Moraes et al. (2004)
		Increase synthesis of collagen fibers	Pinheiro et al. (2004)
		Increase synthesis of elastic fibers	Consonni et al. (2012a)
		Decorin adds	Pinheiro et al. (2005), Pinheiro et al. (2003)
		Enhances expression of MMP-8	Rosa et al. (2011)
D15-18	IpL prolongs and PS separates	Fibroblasts proliferate linearly	Veridiano et al. (2007)
		Structural changes in collagen fibers	Storey (1957), Pinheiro et al. (2004)
		Further synthesis of elastic fibers	Consonni et al. (2012a)
		Versican adds	Pinheiro et al. (2005)
		High molecular weight HA increases	Pinheiro et al. (2005), Rosa et al. (2012)
		Enhances expression of MMP-2 and MMP-9	Rosa et al. (2011)
		RLX rises	O'Byrne and Steinetz (1976), Sherwood (2004)
D18-19	IpL cavity appears PS maximum separation	Fibroblasts proliferation decrease	Veridiano et al. (2007)
		Collagen fiber unraveling	Pinheiro et al. (2004), Pinheiro et al. (2005)
		High molecular weight HA and versican increase	Pinheiro et al. (2005), Garcia et al. (2008)
		Macrophage activation	Castelucci et al. (2020)
		NO enhancement	Moro et al. (2012)
1dpp-40	IpL disappears and PS restores	Cells	Veridiano et al. (2007), Castelucci et al. (2018), Moraes et al. (2004)
		ECM restoration	Consonni et al. (2012a), Rosa et al. (2011)
		Hormone levels drop	Castelucci et al. (2018)
		Macrophages are involved in the repair	Castelucci et al. (2019)

## 2 D1-12

During gestational days 1–12 (D1–12), the microstructure of PS in pregnant mice remained highly consistent with that in non-pregnant mice (Veridiano et al., 2007; Pinheiro et al., 2004). The histological assessment showed that the central fibrocartilaginous disc and bilateral hyaline cartilage layers maintained stable morphology at this stage (Veridiano et al., 2007; Moro et al., 2012), and no significant elastic fiber reorganization or fluctuations in RLX levels were observed (Consonni et al., 2012a; O'Byrne and Steinetz, 1976). Progesterone (P4) supplementation also failed to induce PS structural changes, as confirmed by studies in ovariectomized (ovx) mouse models, suggesting a limited role of hormonal regulation on PS remodeling during this period (HALL, 1956). Based on the above evidence, the present study will not conduct an in-depth mechanistic analysis for this stage.

## 3 D12-15

### 3.1 Separation of PS

Between D12 and D15, the mouse PS undergoes structural expansion from 0.15 mm to 0.2 mm, accompanied by the formation of a distinct IpL (Pinheiro et al., 2004). The IpL originates within PS separation space, characterized by collagen fibers aligned parallel to the ligament’s longitudinal axis and populated by fibroblast-like cells exhibiting proliferative capacity (Moro et al., 2012). This process is accompanied by the proliferation of fibroblasts (Veridiano et al., 2007; Linck et al., 1975), collagen/elastic fiber deposition (Consonni et al., 2012a; Pinheiro et al., 2004), upregulated decorin (small proteoglycan) expression (Pinheiro et al., 2005), and enhanced MMP-8 activity (Rosa et al., 2011). Serum estrogen (E2) levels peak at D14 in pregnant mice (DUONG, 1893). In ovx mice, P4 combined with E2 induces interstitial edema by D12, facilitating early PS expansion (HALL, 1956). This phase marks the initial formation of the IpL and establishes the foundation for subsequent relaxation.

#### 3.1.1 Fibroblast

The IpL gradually replaces the fibrocartilage of the mice between the pubic bones. Proliferative activity in mouse PS cells was quantified through proliferating cell nuclear antigen (PCNA) detection and morphometric analysis, which revealed a gradual increase in cell numbers within the enlarged PS space (Veridiano et al., 2007). Immunohistochemistry and electron microscopy reveal that mouse PS cells are spindle-shaped morphology, embedded within a connective tissue matrix containing collagen and elastin fibers aligned parallel to the pelvic girdle (Consonni et al., 2012a; Veridiano et al., 2007; Moraes et al., 2004). These cells exhibit the classic ultrastructural features of fibroblasts, characterized by a fusiform morphology, smooth nuclear contours, and prominent cytoplasmic organelles (rough endoplasmic reticulum, Golgi apparatus, mitochondria) (Moraes et al., 2004). Notably, phenotypic plasticity observed during this phase suggests adaptive remodeling to accommodate pregnancy biomechanical demands (Sappino et al., 1990).

#### 3.1.2 ECM

The Mouse PS remodeling during gestation involves profound ECM modifications, including collagen reorganization (Pinheiro et al., 2004), elastin network expansion (Consonni et al., 2012a), and proteoglycan/glycosaminoglycan (GAG) composition shifts (Pinheiro et al., 2005; Pinheiro et al., 2003). These changes mitigate compressive stresses during pregnancy and facilitate *postpartum* pelvic stabilization (Pinheiro et al., 2004; Kozel et al., 2006). D12-15, there is the growth of IpL, formed by tightly packed collagen fibrils arranged in fibers distributed along the major axis of the joint, presenting the typical helical organization of collagen crimps (Pinheiro et al., 2004). By immunohistochemical staining, the proteins involved in elastic fiber assembly in IpL have been identified as elastin, fibulin 5, and lysyl oxidation like 1 (LOXL1). During this period, elastic fibers’ shape, length, and diameter have increased, and the expression level of these genes has increased remarkably (Consonni et al., 2012a). The only sulfated GAG on mouse PS is chondroitin sulfate (CS), predominantly contributed by decorin and versican (large proteoglycan) side chains (Rosa et al., 2012). CS/dry weight shows an upward trend in D12-15, presumably due to increased decorin (Pinheiro et al., 2005). MMP-8 expression with collagenase activity is enhanced in D12-15, considered to be associated with IpL formation (Rosa et al., 2011).

### 3.2 Mechanisms related to change

Studies have confirmed that fibroblast proliferation and ECM remodeling play an essential role in forming IpL (Veridiano et al., 2007; Moraes et al., 2004). The formation of collagen fiber and elastic fiber in ECM endows PS with compressive capacity and promotes the formation of IpL. Meanwhile, decorin enhances the pulling force of collagen fibers, and MMP-8 regulates cells and collagen fibers (Balbin et al., 1998). All these promote the remodeling of PS and better maintenance of pelvic stability.

#### 3.2.1 Fibroblast

The IpL fibroblasts exhibit moderate proliferation during D12-15 (Veridiano et al., 2007; Moraes et al., 2004), which may be regulated by the interaction between MMPs and their tissue inhibitors (TIMPs). MMPs regulate cell proliferation and differentiation not only through the degradation of ECM but also via mechanisms such as activating growth factors and modulating their bioavailability (Rosa et al., 2011; Hulboy et al., 1997; McCawley and Matrisian, 2001). Decorin expression is abundant in normal fibroblasts, and versican expression is predominant in hyperplastic fibroblasts (Scott et al., 1995). Although the fibroblasts are proliferative at this time, proliferation is not strong, and decorin is prominently expressed. When D15-18, fibroblasts proliferate strongly, versican is predominantly expressed (Veridiano et al., 2007; Pinheiro et al., 2005). In addition, some cytokines and other components of the connective tissue ECM may also have essential effects on fibroblasts changes (Moraes et al., 2004; Shynlova et al., 2004).

#### 3.2.2 ECM

The first step of elastin assembly is to create elastin aggregates on the surface of fibroblasts (Kozel et al., 2006), which can be assembled by secreting elastin in the cell and producing polymers with reversible deformation and high resilience with the action of enzymes (Consonni et al., 2012a; Kozel et al., 2006). Decorin binds to tropoelastin and fibrillin-containing microfibrils, modulating their assembly and structural integrity (Reinboth et al., 2002). Decorin binding to the d-e bands of type I collagen fibrils plays a crucial role in collagen fibrillogenesis and the regulation of fibril diameter and spacing, contributing to increased tensile stress (Reed and Iozzo, 2002; Scott and Haigh, 1986; Ruhland et al., 2007). When the level of decorin is significantly reduced, collagen fiber defects and instability occur (Markiewicz et al., 2013). Most of the MMP-8 in PS in non-pregnant mice is not activated in chondrocytes (Rosa et al., 2008; Van Lint and Libert, 2006). Between D12 and D15, MMP-8 activity increases, cleaving type I, II, and III collagen fibers into smaller fragments, disrupting collagen structure (Rosa et al., 2011; Balbin et al., 1998; Curry and Osteen, 2003). This, combined with ongoing procollagen I synthesis in the IpL, indicates a high collagen turnover during this stage. Such dynamic remodeling enhances joint flexibility and tissue elasticity, accommodating fetal growth and maternal biomechanical demands (Rosa et al., 2012). Conversely, the decline in MMP-8 activity in late pregnancy (D19) restricts collagen fibers degradation, ensuring the structural integrity of reproductive tissues before parturition (Rosa et al., 2011; Rosa et al., 2008).

## 4 D15-18

### 4.1 Relaxation of PS

The IpL expands from 0.2 mm to 2.4 mm from D15 to D18 (Moraes et al., 2004), and this process is excellent obvious, which is called “relaxation” (Moro et al., 2012; Moraes et al., 2004; HALL, 1947). It includes the proliferation of fibroblasts, the decomposition, untwisting, and dispersion of collagen fibers, and the distribution of water molecules attracted by versican and high hyaluronic acid (HA) in tissues (Pinheiro et al., 2004; Pinheiro et al., 2003; Viell and Struck, 1987; Zhao et al., 2000). These changes ensure that PS can accommodate fetal growth without structural damage.

#### 4.1.1 Fibroblasts

From D15 to D18, the proliferation of cells in PS is intense, with an almost linear increase, with most cells showing myofibroblast-like characteristics, expression of α-smooth muscle actin (α-SMA), and large bundles of intermediate filaments and microfilaments (Veridiano et al., 2007; Moraes et al., 2004). Additionally, junction complexes form between the interior of the cells and the adjacent ECM, which plays a role in transmitting contraction forces within the tissue, helping to support the different mechanical stresses found during pregnancy (Moraes et al., 2004).

#### 4.1.2 ECM

Ultrastructure shows that collagen fibers change their original characteristics after D15; collagen fibers have untwisted, the crimp angles progressively decreased, and the crimp length increased (Pinheiro et al., 2004). Thin wavy elastic fibers randomly distributed in IpL are found by selective staining when elastic fibers’ length and diameter are more pronounced than D12-15 (Consonni et al., 2012a). The CS/dry weight ratio increases at D17-18, presumably due to the rise of versican mRNA expression (Pinheiro et al., 2005; Pinheiro et al., 2003). As a pivotal ECM member, versican provides structural support through its CS chains and synergizes with HA to create hydrated matrices that facilitate PS expansion (Pinheiro et al., 2005; Rosa et al., 2012). The HA probe has the strongest reaction in D18 HA (Rosa et al., 2012). The function of HA depends in part on the size of the molecular weight of HA, a high-molecular-weight polyelectrolyte GAG in mouse PS, which is highly hydrophilic (Rosa et al., 2012), the effect of filling space and promoting tissue hydration and matrix destruction (Jiang et al., 2011).

#### 4.1.3 RLX

RLX is a peptide hormone belonging to the insulin-like growth factor superfamily with two known leucine-rich repeat-containing G protein-coupled receptors (LGRs) named LGR7 and LGR8 (Hsu et al., 2002; Chen et al., 2023). RLX promotes PS expansion in most mammals before parturition (Bedarkar et al., 1977). RLX has different effects in other tissues and is used in anti-fibrosis (Bathgate et al., 2018; Diaz et al., 2020), and regulation of cardiovascular function (Feng et al., 2021; Gao et al., 2019). Studies have shown that serum RLX levels in pregnant mice begin to rise around D12, peak at D18, and decline after parturition (O'Byrne and Steinetz, 1976; Sherwood, 2004). Fibroblast-like cells in the mouse PS express abundant RLX receptors, predominantly LGR7, to which RLX has a high binding affinity (Wang et al., 2009; Yang et al., 1992).

### 4.2 Mechanisms related to change

In a relatively short time, significant changes in PS are caused by specific biochemical processes, especially RLX stimulation and precise regulation of MMPs (Rosa et al., 2011; HALL, 1947). These increase the compliance and extensibility of IpL before delivery.

#### 4.2.1 Fibroblasts

The phenotype transformation of fibroblasts is linked to transforming growth factor-beta (TGF-β) and proteoglycan (Carthy, 2018; Kähäri et al., 1991). MMPs can regulate the bioavailability or activity of growth factors by cleaving matrix and non-matrix substrates or mediating receptor conversion. If MMP-2, MMP-3, or MMP-7 cleaves decorin, TGF-β is released and promotes the induction of the myofibroblast phenotype primarily through activation of the mothers against decapentaplegic homolog (Smad) pathway (Carthy, 2018; Imai et al., 1997). Specifically, Smad2/3 phosphorylation initiates the signaling cascade by enabling direct binding to Smad-binding elements (SBEs) in the promoter regions of target genes, driving the early transcriptional activation of α-SMA and other myogenic proteins (Carthy, 2018). Other studies have shown that HA may co-localize with microtubules and receptor for HA-mediated motility (RHAMM) in mitotic cells, creating an environment conducive to cell division (Hascall et al., 2004). In addition, HA forms hydration zones around cells that promote cell detachment from the matrix, thereby facilitating cell migration and mitosis. This effect is particularly pronounced in late pregnancy and is synchronized with intracellular HA localization during peak cell proliferation (D17- D18) (Toole, 2001; Garcia et al., 2008).

#### 4.2.2 ECM

Myofibroblasts promote synthesizing and secretion in HA and versican (Rosa et al., 2012). Versican and high-molecular-weight HA, as water-holding molecules, are responsible for the hydration of IpL during the third trimester, thereby increasing elasticity (Pinheiro et al., 2005). Moreover, versican and HA may form aggregates (Iozzo, 1998). However, the morphological structure of fibroblasts, along with the physical restriction imposed by collagen and reticular fibers, inhibits the overexpansion of versican and HA (Pinheiro et al., 2005), thereby maintaining tissue structural stability. MMPs with gelatinase activity (MMP-2 and MMP-9) participate in remodeling the basement membrane meshwork in the ECM by degrading type IV collagen fibers (Stygar et al., 2002). The activity of MMPs is tightly regulated by tissue inhibitors of metalloproteinases (TIMPs), with TIMP-1 specifically inhibiting MMP-9, whereas TIMP-2 has a high affinity for MMP-2 (Noda et al., 2003; Nuttall et al., 2004; Gomez et al., 1997). The enzyme spectrum indicates a marked increase in the active forms of MMP-2 and MMP-9 at D15-19, whereas quantitative real-time PCR also reveals high relative expression of TIMP-1 and TIMP-2 during this stage, thus, the dynamic equilibrium between MMPs and TIMPs may play a key role in PS remodeling (Rosa et al., 2011).

#### 4.2.3 RLX

Through specific gene knockout, it has been found that PS can form an early IpL in Rlx−/− female mice but does not relax PS (Zhao et al., 2000; Zhao et al., 1999). Further study finds that the local collagen density of Rlx−/− female mice is too high, and the water content of PS is much less than that of wild-type mice (Zhao et al., 2000). Therefore, RLX may regulate the decomposition and recombination of collagen in target tissues, and increase the concentration of high molecular weight HA, thus promoting PS relaxation and improving compression resistance (Zhao et al., 2000; Kaftanovskaya et al., 2015). Additionally, RLX has regulatory effects on both MMPs and TIMPs. RLX has been shown to stimulate connective tissue remodeling by increasing the expression of MMPs in uterine and cervical fibroblasts and inhibiting collagen synthesis (Arguello-Ramirez et al., 2004; Lenhart et al., 2001). RLX could enhance the expression of TIMP-1 and TIMP-2 in the cervix (Lenhart et al., 2002).

## 5 D18-19

### 5.1 Expansion of PS

IpL is approximately 3 mm on D19 (the day of delivery) (Moraes et al., 2004), and pseudo-cavities appear within IpL (Linck et al., 1976). At this time, PS has maximum expansion and flexibility, contributing to the optimal adjustment of the birth canal and safe delivery. Its physiological process is related to the decomposition and reorganization of collagen fibers (Pinheiro et al., 2004), the increase of hydration capacity of ECM (Pinheiro et al., 2004; Pinheiro et al., 2005), activation of macrophages (Linck et al., 1976), and upregulation of NO (Moro et al., 2012). These changes are essential for enabling PS to withstand the mechanical stresses of labor while maintaining tissue integrity.

#### 5.1.1 ECM

Significant changes occur in various regions of the ECM, particularly in the degradation and remodeling, proportion and arrangement of collagen, molecular changes that promote viscoelasticity, the activity of MMPs, and the increase in GAG (Zhao et al., 2000; Fleischmajer et al., 1991; Neame et al., 2000; Weiss et al., 1979). These combined effects enhance tissue flexibility and improve the ability to resist both tension and compression. In the morphological changes observed in D18 and D19, collagen fibers are separated from fibrils, and the structural cycle of spiral collagen fibers is shortened (Pinheiro et al., 2004; Pinheiro et al., 2005). Collagen fibers change from a dense arrangement to a loose arrangement (Consonni et al., 2012a). By quantitative evaluation of ECM components, it has been found that the gene expression of hyaluronic acid synthase 1, hyaluronic acid synthase 2, and valine increased at D18 (Rosa et al., 2012). HA is 13 times more abundant at D18 than in non-pregnant mice, and the increase stops at D19 (Garcia et al., 2008). Microarray and proteomics analysis of MMP-2 and MMP-9 gene expression and protein production revealed that MMP-2 mRNA and protein levels were significantly upregulated, but MMP-9 mRNA expression was downregulated, and protein production was not detected. However, another study showed that MMP-2 and MMP-9 mRNA expression increased (Rosa et al., 2011). Quantitative real-time PCR shows that TIMP-1 and TIMP-2 are significantly increased at D18-D19 (Rosa et al., 2011), which may prevent excessive tissue damage by MMPs before and after birth and facilitate reasonable relaxation of PS.

#### 5.1.2 Macrophages and NO

Study shows that the number of recruited monocytes is increased in PS and that these recruited monocytes differentiate into pro-inflammatory (M1) or anti-inflammatory (M2) macrophage phenotypes from D18 to 3 dpp, which may contribute to dynamic changes in the gene expression of specific inflammatory mediators involved in PS remodeling at these time points (Castelucci et al., 2019). From D18 to D19, IpL contains non-vascular pseudocavities filled with non-collagenous ECM, mainly composed of mature macrophages (F4/80+) and versican (Castelucci et al., 2020).

NO is a biologically active gas (Imamura et al., 2013) and is produced by NO synthase (NOS) through the oxidation of amide nitrogen of L-arginine (Zeng and Morrison, 2001). It is synthesized by three isomers: endothelial NOS (eNOS), inducible NOS (iNOS), and neural NOS (nNOS) (Su et al., 2005). Some studies have evaluated the morphological, biochemical, and molecular characteristics of iNOS in mouse IpL and found that iNOS is upregulated and NO production is significantly enhanced in chondrocytes and fibroblast-like cells of D19 interpubic tissue (Moro et al., 2012).

### 5.2 Mechanisms related to change

This stage of PS follows the changes of the previous step to promote the relaxation of IpL and smooth parturition. Activation of macrophages (Castelucci et al., 2020), NO increase (Zeng and Morrison, 2001), and proliferation of cells decrease (Veridiano et al., 2007), which is different from the previous stage. The emergence of these new factors suggests that mouse PS undergoes dramatic and complex changes during a brief period of labor, which is fascinating.

#### 5.2.1 ECM

On D18, fibril bundles are assembled to form thin fibers with large spaces between them and some degree of collagen fiber disruption (Pinheiro et al., 2004). RLX stimulates systemic fluid retention (Sunn et al., 2002), while HA functions as a localized molecular sponge within collagen fibrils, thereby promoting tissue hydration essential for interpubic relaxation (Garcia et al., 2008). Versican levels may correlate with F4/80+ cell presence, as activated macrophages secrete versican (Chang et al., 2017). At this stage, increased MMP-2 activity may contribute to the cleavage of versican molecules, as observed in rabbit lung studies (Castelucci et al., 2020; Passi et al., 1999). In addition, the morphology of fibroblasts, collagenous fibers, and reticulum fibers reduces the physical limitation of versican and HA’s complete expansion. The open and highly hydrated ECM contributes to cell migration. The aggregation of macrophages in IpL seems to confirm this (Castelucci et al., 2020). Collectively, the increase of fiber space and the synthesis of high molecular weight HA and versican promote the flexibility and relaxation of IpL (Pinheiro et al., 2005; Garcia et al., 2008).

#### 5.2.2 Macrophages and NO

Macrophages in the IpL of mice exhibit either an M1 (F4/80+/CD40+) or M2 (F4/80+/TfR+) phenotype (Castelucci et al., 2019). M1 macrophages secrete tumor necrosis factor-alpha (TNF-α) and Interleukin-1 alpha (IL-1α), driving sterile inflammation. TNF-α enhances the activity of MMP-2, which degrades versican into damage-associated molecular patterns (DAMPs). These DAMPs, in conjunction with IL-1α, activate Toll-like receptor (TLR) signaling in macrophages, promoting M1 polarization and further recruitment of monocytes (Frey et al., 2013; Schaefer, 2014; Wight et al., 2020). High levels of NO are also markers of M1 activity (Gordon and Taylor, 2005). At this stage, TGF-β secreted by M2 macrophages initiates early repair signaling (Castelucci et al., 2019). We hypothesize that this transition is due to tissue damage in late pregnancy triggering increased vascular permeability and vasodilation, allowing the efficient recruitment of inflammatory monocytes to the injury site. Macrophages predominantly exhibit an M1-like phenotype, producing NO, IL-1α, and TNF-α, which are critical components of antimicrobial immunity (Murray and Wynn, 2011). Additionally, M1 macrophages secrete MMP-2 and MMP-9, which facilitate ECM degradation (Murray and Wynn, 2011). In conclusion, the mouse PS at this stage may be influenced by the differentiation of recruited monocytes and the activation status of macrophages, which may lead to processes associated with an “ordered” inflammatory mechanism (Castelucci et al., 2019).

Studies have found that changes in ECM can promote NO production because ECM changes can enhance the destruction of the actin cytoskeleton, thus increasing the globular actin (G-actin) level (Moro et al., 2012). G-actin also upregulates interleukin-1 beta (IL-1β) to induce iNOS expression, driving the generation of NO (Zeng and Morrison, 2001). NO may modulate the dynamics of α-SMA and desmin, which may help explain the complex adaptations observed in connective tissue cells during relaxation, promoting cytoskeletal alterations (Moro et al., 2012). Other studies believe that the rise of NO at this time might be related to RLX. They find that the generation of NO is parallel to the trend of cyclic RLX (O'Byrne and Steinetz, 1976), and both reach the highest expression at D19 (Varayoud et al., 2001). RLX binds to G protein-coupled receptors (GPCRs), triggering a cascade of 3 ′-5′-cyclic adenosine phosphate (cAMP) activation signals (Halls et al., 2006), which induces the activation of its target NO pathway (Nistri and Bani, 2003) and promotes the production of NO (Nistri and Bani, 2003; Quattrone et al., 2004). LGR7 and LGR8 can also induce the cellular expression of NOS and isoenzyme (Nistri and Bani, 2003), and the expression of RLX receptors in mouse PS fibroblasts is enhanced (Wang et al., 2009), promoting the expression of NO. During pregnancy in mice, RLX is also observed to act directly on smooth muscle by activating NO synthesis *in vivo*. This significantly inhibits ileum movement in mice and affects mesangial cell contraction (Zeng and Morrison, 2001; Vyas-Read et al., 2007). On the other hand, using NOS inhibitors (N^G^-nitro-L-arginine methyl esther) in the middle and late stages of pregnancy has been observed to lead to premature delivery in mice (Tiboni and Giampietro, 2000). Thus, if no direct iNOS are involved in the softening or extension stages, the normal labor activity of mice is affected. It proves that NO has an irreplaceable effect on the relaxation of PS in mice, which is conducive to the optimal regulation of the birth canal and safe delivery (Moro et al., 2012).

D19 cells within the IpL demonstrate the most pronounced cell death phenotype, accompanied by a significant decline in proliferation index. Current evidence suggests that cell death in this context may involve cross-talk between multiple regulated cell death (RCD) modalities, including non-apoptotic pathways such as ferroptosis, autophagy-dependent death, and necroptosis (Veridiano et al., 2007; Zakeri and Ahuja, 1994). However, the precise hierarchy of these pathways and their spatial-temporal coordination require further mechanistic investigation using single-cell sequencing and pathway-specific inhibitors.

## 6 *Postpartum* (1dpp-40dpp)

### 6.1 Recovery of PS and related mechanisms

The interpubic articulation gap of 3dpp is reduced (Consonni et al., 2012a), the hyaline cartilage cap of 10dpp is restored (Castelucci et al., 2018), and the similar original shape of 40dpp is restored (Consonni et al., 2012b). This phenomenon is formerly known as“PS metamorphosis” (WU, 1936). Tissue remodeling in the two stages of IpL degradation and fibrochondral tissue recovery involves significant changes in ECM and interpubic cells (Veridiano et al., 2007; Pinheiro et al., 2003). This process is crucial for restoring the structural integrity and functionality of PS.

#### 6.1.1 Cells

*Postpartum* fibroblasts have a myofibroblast-like phenotype and are distributed along collagen fibers (Moraes et al., 2004). This phenotypic change may be associated with macrophages; macrophage-derived TGF-β contributes to tissue regeneration and wound repair by promoting fibroblast differentiation into myofibroblasts (Murray and Wynn, 2011; Desmoulière et al., 2005). It is speculated that myofibroblasts can help the birth canal close following delivery by pulling the pelvic bones together (Moraes et al., 2004). Angular chondrocyte-like cells increase primarily in the bone distal region of the IpL osteoligamentous junction at 5dpp and hyaline cartilage at 10dpp. This is necessary to restore PS hyaline cartilage cap (Castelucci et al., 2018).

Colocalization of *postpartum* F4/80+ cells with HA aligns with the presence of M2 macrophages (F4/80+/TfR+) and the high expression levels of the interleukin-10 (*Il10*) gene in the pubic symphysis tissue (Castelucci et al., 2019). IL-10 inhibits TNF-α and IL-1α, while HA suppresses TLR signaling, thereby blocking DAMP-driven M1 polarization. At this stage, the complement system shifts from complement component 3a (C3a, pro-inflammatory) to component 1, q subcomponent (C1q, pro-repair), promoting the “silent phagocytosis” of apoptotic cells and preventing immune activation (Castelucci et al., 2019; Castelucci et al., 2020; Egami, 2016). It is well established that once inflammatory stimuli or pathogens are cleared, M1 activation subsides, and the immune response transitions into a wound-healing phase characterized by the accumulation of M2 macrophages (Murray and Wynn, 2011). Therefore, macrophage activation and polarization facilitate the efficient recovery and repair of PS after birth, thereby ensuring the mechanical stability of the reproductive tract and its capacity to initiate and sustain subsequent pregnancies (Castelucci et al., 2020).

#### 6.1.2 ECM

Elastic fiber synthesis and assembly are critical to restoring pelvic organ support after vaginal delivery. Studies have found that elastic fiber homeostasis disorder is the main event in mice’s pathogenesis of pelvic organ prolapse (Drewes et al., 2007). Elastin is the substrate of lysyl oxidase (LOX) and LOXL1, which is essential to ensure elastic fibers’ homeostasis and elasticity (Liu et al., 2004; Noblesse et al., 2004). Meanwhile, fibulin-5 (FBLN5) plays an active role in the correct folding of elastin (Drewes et al., 2007). The relative gene expressions of proelastin mRNAs, fibulin-5 (FBLN5), and LOXL1 in PS tissues increased, and elastic fiber length increased in 1dpp. After 3dpp, elastic fiber length is shortened, reaching the level of non-pregnant mice (Consonni et al., 2012a). From D19 to 1dpp, ECM is reabsorbed in the bone distal region of the IpL osteoligamentous junction (Castelucci et al., 2018). After 5dpp, ECM deposition in hyaline cartilage gradually increases and returns to a normal level at 10dpp (Castelucci et al., 2018). HA decreases from D19, and the HA at 5dpp is similar to that of D12 (Garcia et al., 2008). *Postpartum* MMP-2 and MMP-9 gradually decrease, and MMP-9 at 5dpp returns to non-pregnant levels (Rosa et al., 2011).

#### 6.1.3 Hormones

It has been reported that the serum levels of E2, P4, and RLX in *postpartum* mice are relatively low compared with the end of pregnancy (Ruth, 1935; Bedarkar et al., 1977; Parry and Vodstrcil, 2007). Low E2 levels allow for interaction with factors that bind to the promoter of type II collagen (COL2A1) and SRY-related high-mobility group-box 9 (SOX9), which can enhance its expression of undifferentiated articular chondrocytes and thus drive its differentiation to maturity. During this period the differentiation of osteochondral progenitor cells and proliferation of differentiated chondrocytes at PS in mice is conducive to the recovery of fibrocartilage disc and hyaline cartilage cap (Castelucci et al., 2018).

## 7 Discussion

The mouse PS is essential for maintaining pelvic stability while allowing dynamic adaptation during parturition. This structure undergoes a precisely orchestrated physiological cascade involving stage-specific ECM reorganization, cellular phenotypic modulation, hormonal fluctuations, and immune cell involvement. This review delineated the mechanistic interplay underlying mouse PS transformation, emphasizing mid-to-late gestational remodeling and *postpartum* recovery.

Mice serve as an important model for studying childbirth-related processes, offering insights that are often difficult to obtain from human studies due to ethical and practical limitations (Ratajczak and Muglia, 2008). Both mice and humans exhibit fibrocartilage-to-IpL transitions during pregnancy, enabling PS expansion (Ortega et al., 2003; Becker et al., 2010). However, there are differences between humans and mice, such as variations in RLX levels. In pregnant mice, serum RLX levels begin to rise around D12 and peak at D18 (O'Byrne and Steinetz, 1976). In contrast, pregnant women experience an initial increase in RLX levels until they peak at approximately 12 weeks of gestation, followed by a decline and stabilization around 17 weeks (Kristiansson et al., 1996). This temporal disparity suggests species-specific windows of RLX-mediated tissue plasticity: predominantly mid-late gestation in mice *versus* first and second trimesters in humans. In guinea pigs, the IpL demonstrates substantial neovascularization preceding parturition (Rodríguez et al., 2003). Certain bat species exhibit remarkable IpL expansion, achieving dimensions comparable to or exceeding the pelvic canal’s maximum transverse diameter (Grunstra et al., 2019). Conversely, rats retain hyaline cartilage without IpL formation throughout reproduction (Ortega et al., 2003). These species-specific variations in PS responses underscore evolutionary adaptations optimized for distinct reproductive strategies.

In summary, the remodeling and relaxation of the PS during pregnancy is a highly regulated and essential process for successful parturition. Studies in animal models, particularly mice, have provided significant insights into the cellular and molecular mechanisms involved in PS adaptation, ECM remodeling, cell proliferation, and hormonal signaling. Future investigations should prioritize multi-omics approaches to resolve these complexities. Spatial transcriptomics could map microdomain-specific gene expression patterns during PS transformation, while single-cell proteomics may delineate hormone-responsive cell subpopulations. Meanwhile, advanced imaging modalities, such as *in vivo* micro-CT with contrast-enhanced visualization of ligamentous structures, would enable dynamic tracking of architectural changes. These approaches are necessary to fully elucidate the mechanisms underlying PS remodeling and to enhance our understanding of reproductive physiology.
